# Identification of Lysine Misincorporation at Asparagine Position in Recombinant Insulin Analogs Produced in ***E. coli***

**DOI:** 10.1007/s11095-019-2601-z

**Published:** 2019-04-04

**Authors:** Dorota Stadnik, Anna Bierczyńska-Krzysik, Joanna Zielińska, Jarosław Antosik, Piotr Borowicz, Elżbieta Bednarek, Wojciech Bocian, Jerzy Sitkowski, Lech Kozerski

**Affiliations:** 1 Łukasiewicz Research Network – Institute of Biotechnology and Antibiotics, Starościńska 5, 02-516 Warsaw, Poland; 2 National Medicines Institute, Chełmska 30/34, 00-725 Warsaw, Poland

**Keywords:** insulin analogs, insulin impurities, mass spectrometry, misincorporation, NMR

## Abstract

**Purpose:**

Identification of human insulin analogs’ impurity with a mass shift +14 Da in comparison to a parent protein.

**Methods:**

The protein sequence variant was detected and identified with the application of peptide mapping, liquid chromatography, tandem mass spectrometric analysis, nuclear magnetic resonance spectroscopy (NMR) and Edman sequencing.

**Results:**

The misincorporated lysine (Lys) at asparagine (Asn) position A21 was detected in recombinant human insulin and its analogs.

**Conclusions:**

Although there are three asparagine residues in the insulin derivative, the misincorporation of lysine occurred only at position A21. The process involves G/U or A/U wobble base pairing.

**Electronic supplementary material:**

The online version of this article (10.1007/s11095-019-2601-z) contains supplementary material, which is available to authorized users.

## Introduction

Human insulin is a protein hormone produced in pancreatic beta cells. In type 1 and sometimes also in type 2 diabetes, insulin must be administered exogenously. Contemporary preparations are based on recombinant human insulin or its analogs produced by rDNA technology (1,2). These proteins are biosynthesized in *E. coli* or yeast as a precursor polypeptide which is then cleaved by trypsin and carboxypeptidase B (CPB) to achieve a two-chain protein (3). Prior to formulation into the final dosage forms, the desired protein must be extensively purified to remove process- and product-related impurities (4). Process-related impurities include: cell culture media, host cell proteins, DNA, chromatographic media used in purification, solvents and buffer components (5). Product-related impurities result from post-translational modifications and degradation events induced by the manufacturing process conditions. Properties of these modified entities are different from those of the desired protein and may affect safety and efficacy of a drug product (5). The best known impurities of insulin and insulin analogs are desamido A21 and desamido B3 derivatives (6). Other related proteins of insulins identified during drug production or storage are: des-Thr-insulin which is an undesirable side-product, di-Arg^B31-B32^-insulin, and Arg-insulin, which are intermediates during insulin production, *N* αB1 carbamoyl insulin, *N* αA1 carbamoyl insulin (7–9) acetylated lispro insulin (10), desPhe^B1^-N-oxalyl-Val^B2^ insulin (11), desPhe^B1^-, desPhe^B1^-N-formyl-Val^B2^- and pyroGlu^B4^ insulin (12), covalent insulin dimers (13), insulin fragments (14), misincorporated A9 (Ser → Asn) human insulin (14) and insulin with amino acid residues oxidized to 3,4-dihydroxyphenylalanine (DOPA) and 2-amino-3-(3,4-dioxocyclohexa-1,5-dien-1-yl) propanoic acid (DOCH) (15). Such impurities present in a protein substance and dosage forms cannot exceed acceptable amounts in order to meet the Pharmacopoeia requirements (16).

Increasing requirements for purity of biotechnological products force manufacturers to reduce unwanted impurities to the greatest possible extent. Therefore isolation and identification of protein impurities in biotechnological products is a challenge because of their low content in a drug substance or in a formulation. Multiple chromatographic separations and pooling of desired fractions are applied in order to collect a sufficient amount of material for further identification and characterization (11). This is often preceded by keeping a material under stress conditions in order to increase the content of impurities of interest (13).

Nowadays, mass spectrometry is the first method of choice for identification of posttranslational modifications (PTMs) in proteins (17,18). The procedure involves enzymatic protein digestion and analysis of the resulting peptides, usually after enrichment, by tandem mass spectrometry (MS/MS). The type of modification and its site is inferred based on the mass shift detected in the precursor ion and in the fragment ions bearing the modified amino acid residue. Problems can occur when different PTMs have almost the same mass shift or when it comes to the distinction between chemical modifications and amino acid misincorporations of the same or similar mass (19,20). The latter is even more challenging as amino acid misincorporations are less frequently detected and reported than PTMs. High accuracy mass spectrometry with an advanced in-house software for acquisition and data analysis is necessary for confident identification of modified amino acids in proteins (19). Manual inspection of MS/MS spectra is required in distinguishing modifications of similar mass. For example, a tri-methylated and acetylated lysine residue differ by 0.0363 Da. Only the unique immonium ions at 126.1 amu in fragmentation patterns were found to distinguish the two modifications from each other (21). However unique diagnostic ions in fragmentation patterns are not common for all PTMs and additional analytical techniques are required for unequivocal identification.

In this paper, we describe the identification of an insulin analogs’ impurity with a mass shift +14 Da in comparison to a parent protein. Based on mass spectra of an intact molecule, polypeptide chains and enzymatic digests this impurity was suspected to be a methylated derivative resulting from post-translational modification. Additional techniques, MS/MS fragmentation, NMR and Edman sequencing, have revealed that lysine was misincorporated at an asparagine A21 position resulting in +14 Da mass difference.

## Experimental Section

### Chemicals and Materials

All chemicals were of analytical reagent grade. Hydrochloric acid 35–38% and acetonitrile were purchased from Avantor (Gliwice, Poland). Glacial acetic acid, sodium hydroxide, phosphoric acid 85%, iminodiacetic acid, arginine, urea, HEPES, dithiothreitol (DTT), iodoacetamide (IAA), 1,5-diaminonaphthalene (DAN), formic acid were purchased from Sigma-Aldrich (Munich, Germany). Trifluoroacetic acid (TFA), ammonium sulfate, ammonium carbonate, 2-propanol were purchased from Merck (Darmstadt, Germany). Pharmalyte 3–10 carrier ampholytes were from GE Healthcare (Chicago, Illinois). Gel and peptide markers for cIEF were from Beckman Coulter (Brea, California, USA). Endoproteinase Glu-C (Protease *S. aureus* V8) was from Thermo Scientific (Waltham, Massachusetts, USA). Trideuteroacetonitrile (CD3CN, 100 atom % D) was purchased from ARMAR Chemicals, (Döttingen, Switzerland). 3-(trimethylsilyl) 3,3,2,2-tetradeuteropropionic acid sodium salt (TSPA-d4 purity: 96.9%) was purchased from Dr. Glaser AG (Basel, Switzerland). Carboxypeptidase B (CPB) was from Roche (Basel, Switzerland). Lys^B31^Arg^B32^ human insulin, insulin lispro precursor, Ala^A22^Lys^B31^Arg^B32^ human insulin, Gly^A22^Lys^B31^Arg^B32^ human insulin were from the Institute of Biotechnology and Antibiotics (Warsaw, Poland). Insulin and analogs were produced by recombinant DNA technology in *E. coli* (K12) essentially as described previously (22).

### Isolation of Impurity KR14

The impurity was isolated by reverse phase HPLC on a Waters model 600 system (Waters, Milford Massachusetts, USA) with UV detection at 214 nm, equipped with a Kromasil C8, 100 Å, 13 μm column, 21.2 × 250 mm (Sigma-Aldrich, Munich, Germany). Mobile phase was: A: 0.02 M ammonium sulfate, pH 2.7, 15% (*v/v*) 2-propanol, B: 0.02 M ammonium sulfate, pH 2.7, 50% (*v/v*) 2-propanol. Elution was carried out at a low rate 4 ml/min using a gradient of 15–40% B over 2 h. The resulting fractions containing KR14 were desalted, lyophilized and stored at −20°C.

### Preparation of Polypeptide Chains a and B

Impurity KR14 samples were dissolved in 0.1 M ammonium bicarbonate, reduced with DTT and alkylated with IAA. Then the A and B chains of impurity KR14 were separated using the HPLC system (Waters Alliance 2695, Milford, USA) equipped with a Zorbax SB-C18 1.8, 50 mm × 4.6 mm column (Agilent, Santa Clara, CA, USA). The separation was carried out at 40°C with a linear gradient elution from 10% to 50% eluent B in 20 min at a flow rate 1 ml/min. Eluent A was 0.1% TFA and eluent B was 0.1% TFA with 90% ACN (both vol/vol). The fractions containing the A and B chains were lyophilized and stored at −20°C.

### Enzymatic Digestion with Endoproteinase Glu-C

Impurity KR14 samples were dissolved in 0.1 M HEPES buffer pH 7.5 and mixed with endoproteinase Glu-C in 10:1 (*w/w*) ratio. The mixture was incubated for 4 h at 37°C, and the reaction was stopped by acidification with formic acid. Then the HPLC system (Alliance 2695, Waters, Milford, USA) equipped with a Spherisorb ODS2 column C18, 3 μm, 4.6 × 100 mm and UV detector set at 214 nm was employed to separate the peptides. The separation conditions were as described in European Pharmacopoeia, monograph 01/2011:0838 (16), peptide mapping. The digest was also analyzed on a 4800 MALDI-TOF/TOF spectrometer (Applied Biosystems, Framingham, USA).

### Isolation of Peptide A18-A21/B14-B21

Impurity KR14 was digested with endoproteinase Glu-C as mentioned above. The HPLC system (Alliance 2695, Waters, Milford, USA) equipped with a Zorbax SB-C18 1.8, 50 × 4.6 column (Agilent, Santa Clara, California, USA) and UV detector set at 214 nm were employed to separate and isolate the peptide A18-A21/B14-B21. The separation was carried out at 40°C with a linear gradient elution from 10% to 50% eluent B in 20 min at a flow rate 1 ml/min. Eluent A was 0.1% TFA and eluent B was 0.1% TFA with 90% acetonitrile (ACN). The fraction containing peptide A18-A21/B14-B21 was collected with Fraction Collector III (Waters, Milford, USA) and the eluate was evaporated as mentioned above.

### Preparation of Peptide A18-A21 for MS/MS Fragmentation

The fraction containing peptide A18-A21/B14-B21 was dissolved in 0.1 M ammonium bicarbonate, reduced with DTT and alkylated with IAA. The samples after reduction and alkylation were stored at −20°C until MS/MS analysis.

### Enzymatic Digestion with Carboxypeptidase B

Impurity KR14 was dissolved in 0.01 M HCl to the concentration of 4 mg/ml. The protein solution was mixed with 0.1 M HEPES buffer pH 8.5 and 5 mg/ml carboxypeptidase B in 10:1 (*w/w*) ratio. Similarly, the peptide A18-A21/B14-B21 was dissolved in 0.1 M HEPES buffer pH 8.5 and mixed with 5 mg/ml carboxypeptidase B in 10:1 (*w/w*) ratio. Both mixtures were incubated for 1 h at 37°C. The reaction was stopped by acidification with formic acid.

### MALDI-TOF/TOF Mass Spectrometry and Data Acquisition

Molecular weight measurements were performed with 4800 MALDI-TOF/TOF Analyzer (Applied Biosystems, Framingham, USA). The mass spectrometer equipped with a 200 Hz, 355 nm Nd:YAG laser operated in positive ion reflector mode. The acceleration voltage was set to 20 kV, lens to 10 kV and the delayed extraction time to 250 ns. Each mass spectrum was obtained by the accumulation of 1024 laser shots. Alpha-Cyano-4-hydroxy-cinnamic acid (Sigma-Aldrich, Munich, Germany) dissolved in 50:50 water/acetonitrile (J.T. Baker, Deventer, The Netherlands) with 0.1% TFA – final concentration (Sigma-Aldrich, Munich, Germany) was the matrix used. The instrument was calibrated with a 4700 proteomics analyzer calibration mixture provided by Applied Biosystems. Acquired spectra were processed with Data Explorer Software, Version 4.9 (Applied Biosystems, Framingham, USA). Fragmentation spectra were performed for the selected precursor ions detected in the MS scan using air as the collision gas and a collision energy of 1 kV. The instrument was calibrated using the fragmentation spectrum of Glu1-Fibrinopeptide B (EGVNDNEEGFFSAR). The MS/MS spectra resulted from the accumulation of 2000 laser shots.

### NMR Experiments

The preparation of the NMR sample was conducted by the similar procedure, as described by Hua *et al* (23) except that H_2_O/CD_3_CN (73/27 vol%) was used as a solvent in this study. The protein solution concentration was 2.9 mM, pH 2.3 was adjusted by adding the aliquots of HCl or NaOH solutions. The ^1^H and ^13^C NMR spectra were calibrated *vs*. TSPA-*d*_4_. For the ^15^N chemical shifts, liquid NH_3_ was used as an external reference according to IUPAC recommendation.

NMR spectra were run at 298 K on a Varian VNMRS 500 MHz spectrometer (Varian, Palo Alto, California, USA) equipped with 5-mm Z-SPEC Nalorac IDG500-5HT probe (Nalorac Corp., Martinez, California, USA) with an actively shielded z-gradient coil.

NOESY spectrum (24) as DPFGSE-NOESY experiment with water suppression by gradient echo was recorded using the States-TPPI method (25, 26) to obtain phase-sensitive data, with a 6000 Hz sweep width in both dimensions, 2 K complex data points in *t*_2_, 16 transients with 512 complex data points in *t*_1_, a 200 ms mixing time and relaxation delay of 2 s. Spectrum was apodized in both dimensions using a sq-cosine function. The linear prediction was applied in *t*_1_ to extend the data twice, with zero filling to yield 2 K complex data points.

TOCSY spectrum (27, 28) (WGTOCSY with selective H_2_O one-lobe sinc pulse with flipback) was acquired with a 6000 Hz sweep width in both dimensions, 2 K complex data points in *t*_2_, 64 transients with 512 complex data points in *t*_1_with relaxation delay of 1.5 s. The mixing time for TOCSY spectrum was 90 ms with a DIPSI-2 spin-lock field of 7 kHz. Spectrum was apodised in both dimensions using a sq-cosine function. The linear prediction was applied in *t*_1_ to extend the data twice, with zero filling to yield 2 K complex data points.

C2hsqcse (29–31) sensitivity-enhanced phase-sensitive HSQC experiment with complex adiabatic pulses and broadband inversion pulses (Bip). The ^1^H-^13^C HSQC NMR spectra were obtained with a spectral width of 5000 Hz, 1 K complex data points in *t*_2_ and 8000 Hz, 128 transients with 512 complex data points in *t*_1_ with a relaxation delay of 1 s and ^1^*J*(C, H) = 135 Hz. The ^1^H-^15^N HSQC: spectral width of 6000 Hz, 1 K complex data points in *t*_2_ and 2500 Hz, 256 transients with 200 complex data points in *t*_1_, with a relaxation delay of 1 s and ^1^*J*(N, H) = 86 Hz. The linear prediction was applied in the *t*_1_ dimension to extend the data twice, with zero filling to yield 2 K complex data points.

### N-Terminal Sequencing

Determination of N-terminal sequence of fragment II of impurity KR14 by Edman degradation was performed on a Procise 491 (Applied Biosystems, Framingham, USA) automatic protein sequencer. Before analyses, the instrument was calibrated using a commercial PTH proteinaceous amino acid standard mixture (Wako, Osaka, Japan). The sequencing was done using polypeptide chains absorbed on TFA-treated glass fiber disks (Wako, Osaka, Japan) coated by polybrene (Sigma-Aldrich, Munich, Germany).

## Results and Discussion

### Localization of Modification Site

Recombinant human insulin and its analogs are produced at IBA by the precursor method using *E. coli* expression system by the similar procedure, as described in a literature (22). One- or two-step enzymatic digestion is applied depending on the type of targeted protein (Fig. S1 in Supplementary materials). An extensive multi-step purification is performed to remove undesired impurities to achieve >98% purity of a drug substance. During development and optimization of the manufacturing process, we were interested not only in high purity of the desired recombinant proteins but also in the identification of impurities – related proteins which are formed through the process. Because the content of related proteins in a final drug substance was very low (any impurity: maximum 0.4%, total impurities: maximum 2.0%), scouting experiments were performed on waste fractions collected during the purification. Small aliquots of collected fractions were subjected to MALDI-TOF MS profiling to detect protein impurities. The selected fractions were additionally purified and concentrated for further investigation.

**Fig. 1:**
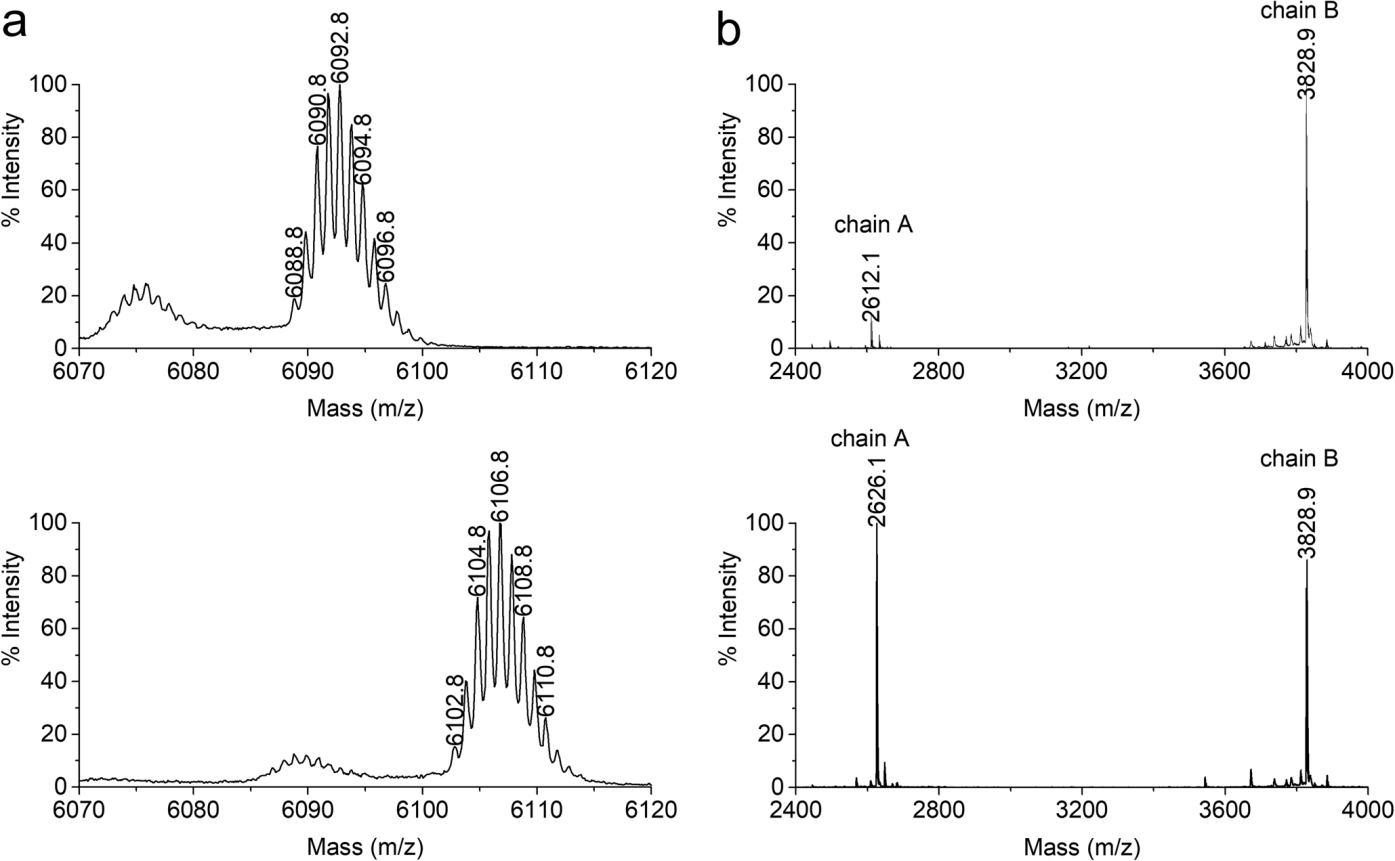
MALDI-TOF spectrum of KR (upper) and impurity KR14 (bottom) **a**) the intact molecules, **b**) chains A and B after reduction and alkylation of intact molecules

In scouting experiments, we have found an impurity with a mass increased by 14 Da compared to the mass of Lys^B31^Arg^B32^ human insulin (named hereinafter insulin KR) – our first long-acting insulin analog (32) and an intermediate in the production of human insulin (Fig. S1 in Supplementary materials). The calculated and obtained monoisotopic mass of insulin KR was 6088.8 Da whereas the mass of the impurity, named hereinafter KR14, measured by MALDI-TOF MS was 6102.8 Da as shown in Fig. 1a. Considering the mass shift value of 14 Da, methylation was herein the most suspected modification. The exact localization of the modification site was established by evaluation of the peptide chains m/z values and peptide mapping.

First, the impurity KR14 was reduced with DTT and alkylated with IAA. The resulting polypeptide chains A and B were analyzed using MALDI–TOF MS. Insulin KR treated in an analogous manner was used as a reference. Figure 1b shows that the spectra of the B chain for the impurity KR14 and the reference are compatible, whereas the spectra of the A chain revealed 14 Da mass shift of impurity KR14 in comparison to the reference spectrum of insulin KR. This provide a clear evidence that the modification occurred in the A chain.

**Fig. 2:**
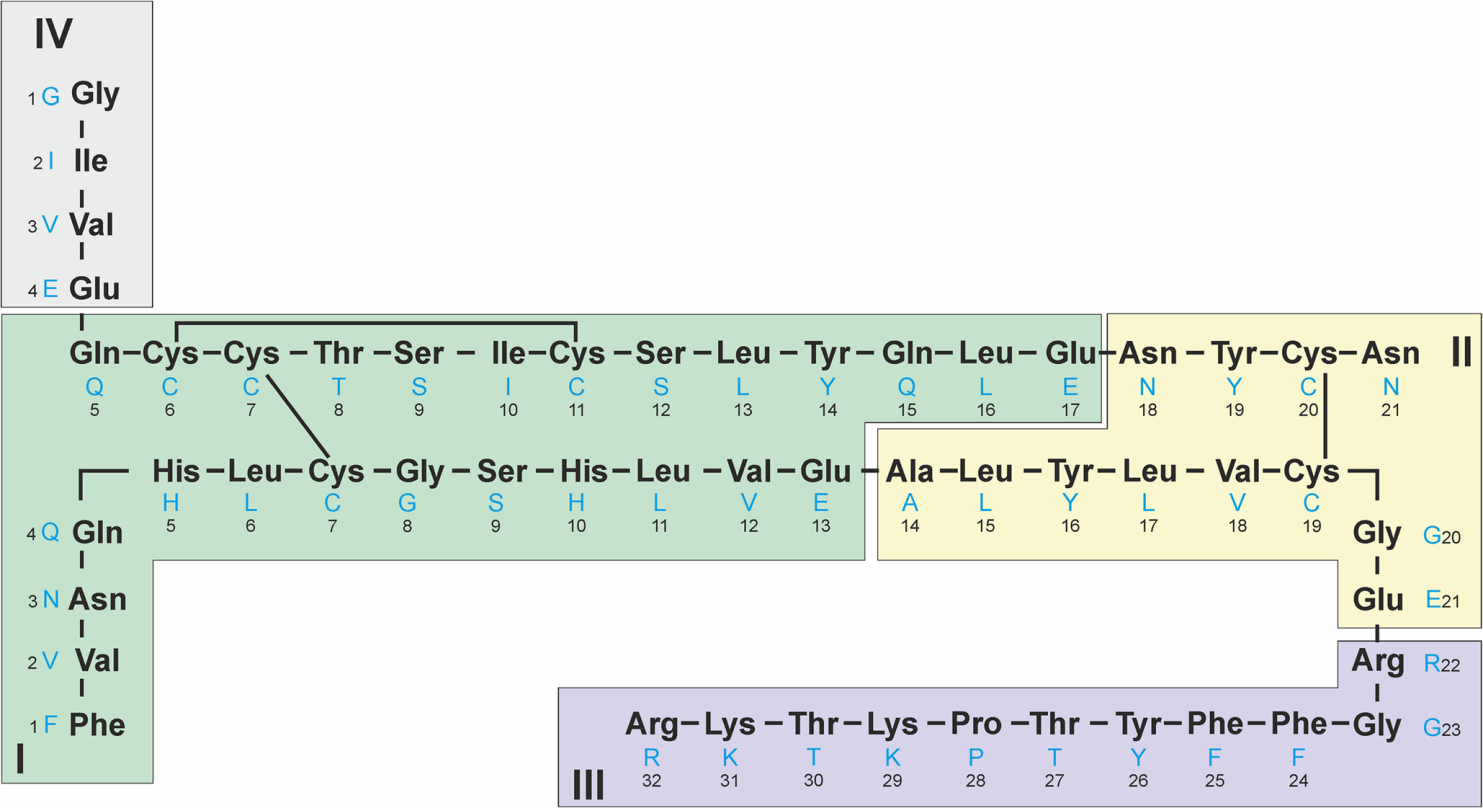
Insulin KR fragments resulting from enzymatic digestion with endoproteinase Glu-C

For more accurate localization of the modification site, the intact impurity KR14 was consequently digested with endoproteinase Glu-C. The same digestion procedure was applied simultaneously to human insulin KR which was used as a reference. Endoproteinase Glu-C selectively cleaves insulin KR into four fragments, two of which comprise disulphide bonds (Fig. 2).

**Fig. 3:**
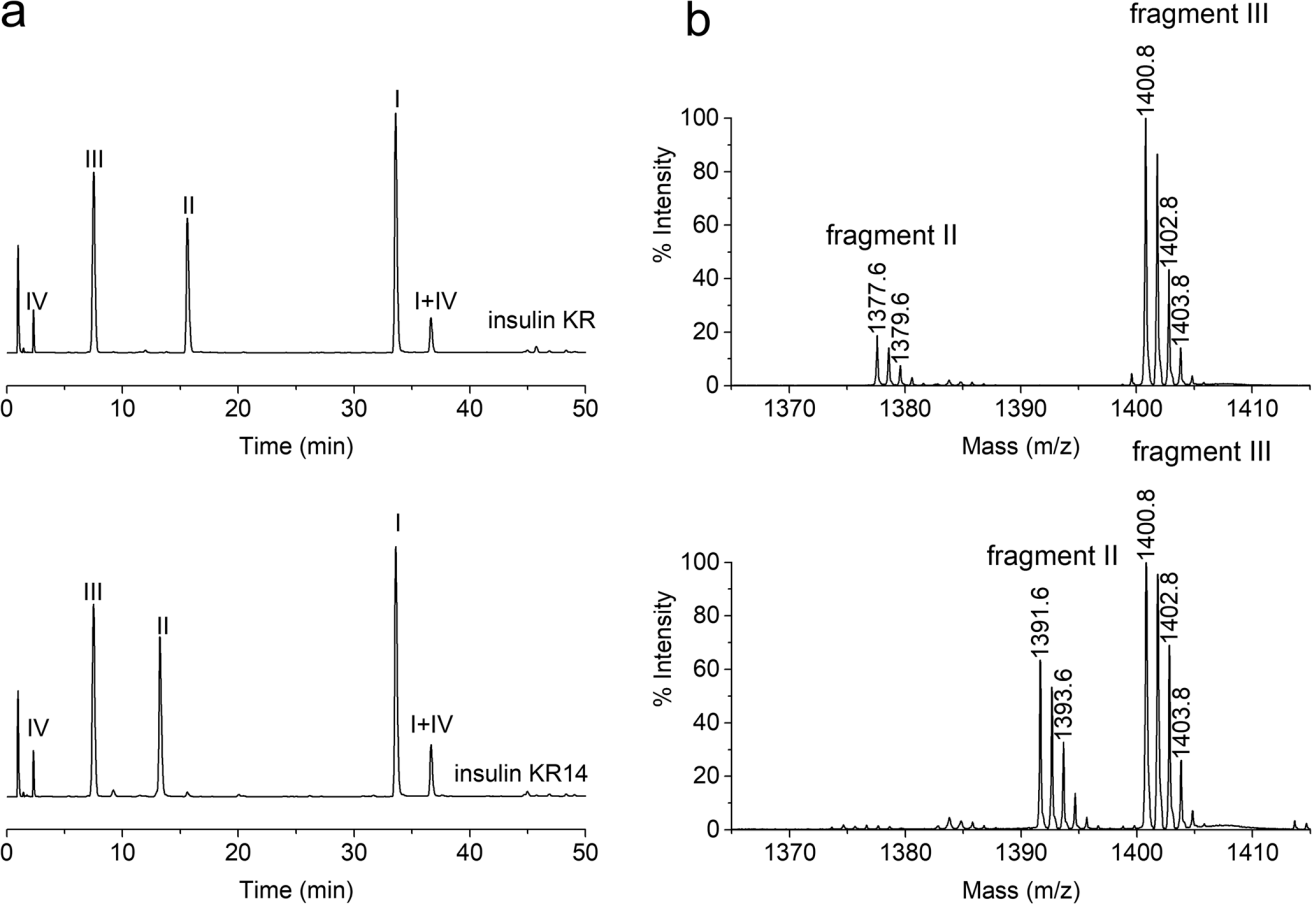
Peptide map of insulin KR (upper) and impurity KR14 (bottom) after enzymatic digestion with endoproteinase Glu-C **a**) chromatogram RP-HPLC-UV, **b**) MALDI-TOF spectrum – fragments II and III are shown

The digests were analyzed by RP-HPLC UV and MALDI-TOF MS. As shown in Fig. 3a, retention times of the peptide fragments I, III, IV are consistent for the reference and impurity KR14 but there is a significant difference in retention times of the corresponding fragments II. Considering this and the results obtained for the polypeptide chains presented above, it became clear that the modification was within peptide A18-A21. Similarly to RP-HLC results, the presented spectra (Fig. 3b) are consistent for the reference insulin KR and impurity KR14 with one exception related to fragment II (A18-A21). For the analyzed derivative, this peptide appears with a + 14 Da mass shift.

**Fig. 4:**
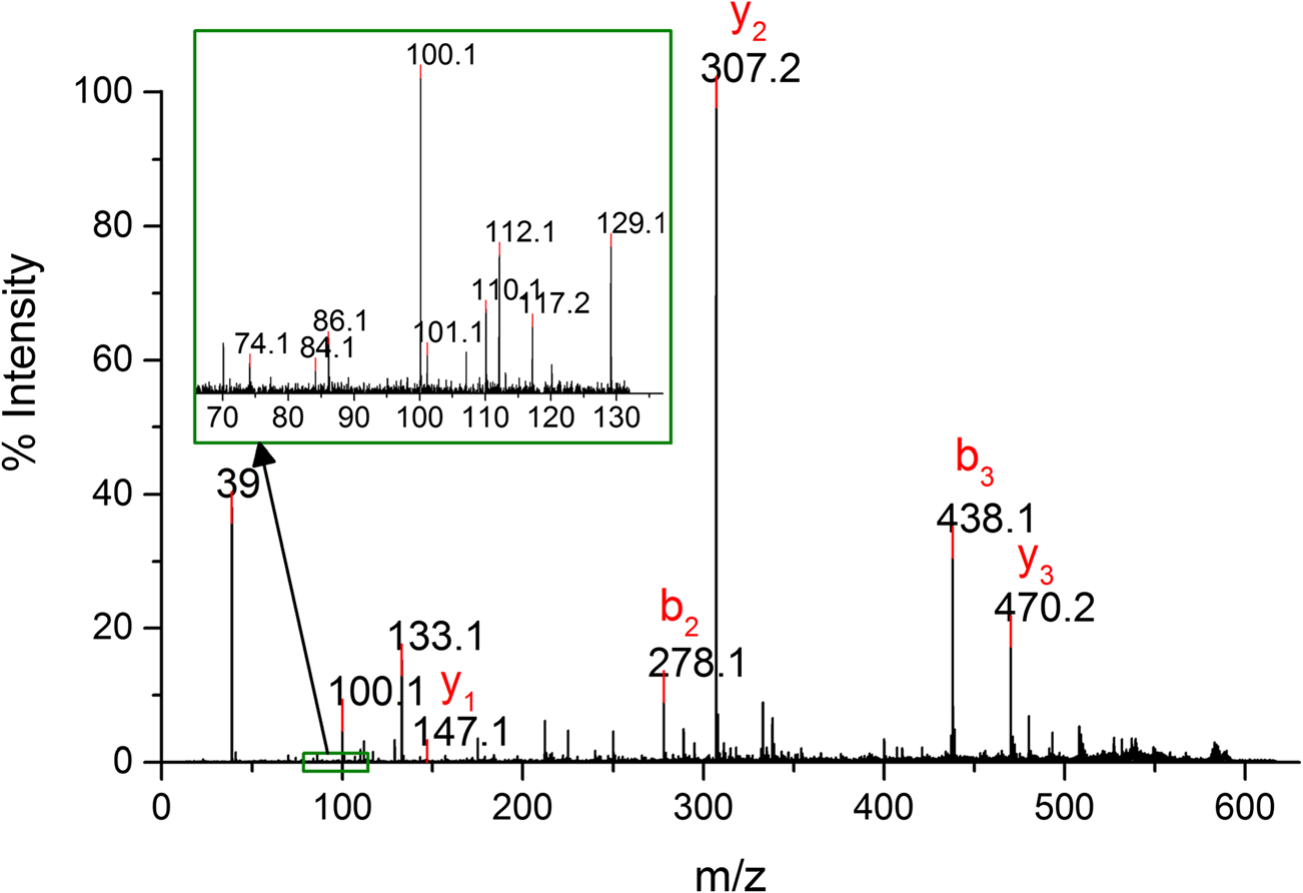
MALDI-TOF/TOF spectrum of peptide A18-A21 resulting from impurity KR14

To unambiguously assign the modified residue in peptide A18-A21, the impurity KR14 digest (after reduction and alkylation) was subjected to MALDI-TOF/TOF fragmentation analysis. Fragmentation of peptide A18-A21 resulted in two b-ions, b2 and b3, and three y-ions (Fig. 4). The b2 and b3 ions did not contain any modification and correspond to the unmodified sequence NYC in tetrapeptide A18-A21. The y-type ions all were shifted by 14 units. Based on these data, it was concluded that only the C-terminal amino acid residue in the tetrapeptide was altered by 14 Da moiety.

The very first post-translational modification with 14 Da mass increase that comes to mind is methylation. Although this modification was mainly described for lysine and arginine residues in proteins (33, 34), methylation of asparagine residue was also documented (35). Apart from methylation of asparagine, misincorporation of lysine or glutamine for asparagine must be also considered for +14 Da mass shift. The suspicion that residue A21 was methylated asparagine became questionable after careful inspection of MSMS spectrum in Fig. 4 (see the inset in Fig. 4), where immonium and related ions 74, 84, 101, 129 were detected. The ions at m/z 84, 101, 129 in fragmentation pattern are characteristic for lysine and glutamine residues (36, 37) but the lack of the ion at m/z 75 and presence of the ion at m/z 74 is indicative of lysine. These two ions distinguish Lys from Gln in fragmentation patterns of amino acids obtained by MALDI-TOF/TOF tandem MS (38). Another evidence of Lys misincorporation at position 21 was sought from the additional experiment, including digestion with CPB and mass spectrometry analysis.

CPB is an enzyme that catalyzes the hydrolysis of the basic amino acids, lysine, arginine from the C-terminal position of polypeptide chains and therefore enables differentiation between Lys and Gln in KR14. Digestion was performed on intact impurity KR14 concurrently with the reference insulin KR and on the isolated fragment II.

The data presented in the Supplementary Material (Digestion with CPB, Fig. S2, S3, S4) indicate that the KR14 derivative has Lys at position A21, but this residue is not digested by CBP in the intact protein probably due to structural hindrances. In order to ultimately verify whether the obtained data indeed corresponded with misincorporated Lys at position A21, two additional techniques were applied which included automated Edman sequencing and NMR spectroscopy.

### Elucidation of the Structure of the Impurity KR14 by Edman Degradation and NMR Analysis

Fragment II of impurity KR14 was isolated from the digest and analyzed by automated Edman degradation. This fragment consists of A18-A21 residues of the A chain and B14-B21 residues of the B chain connected with one disulphide bond between A20-B19. Therefore two amino acids were released during each cycle of the Edman degradation. In the 4th cycle related to residues A21 and B17 in fragment II, Leu and Lys were detected simultaneously (Fig. S5 in Supplementary materials). Based on the theoretical sequence of fragment II, Leu was assigned to B17 whereas Lys was assumed to originate from residue A21.

To confirm the existence of lysine at A21 in the impurity KR14, a complete analysis of TOCSY, ^1^H/^13^C-HSQC and NOESY spectra were performed on the undigested impurity KR14 in comparison to insulin KR. The assignments of proton signals and carbon atom signals to the corresponding CH, CH_2_ or CH_3_ groups were confirmed by the ^1^H/^13^C-HSQC spectrum. Then the NOESY spectrum was analyzed to allow for the unambiguous assignment of all amino acids to a specific site in the A or B chain of the impurity KR14 (primary structure). Additionally, the ^1^H/^15^N-HSQC spectrum was measured and the nitrogen atoms chemical shifts of the amide groups from the main chains were determined.

**Fig. 5:**
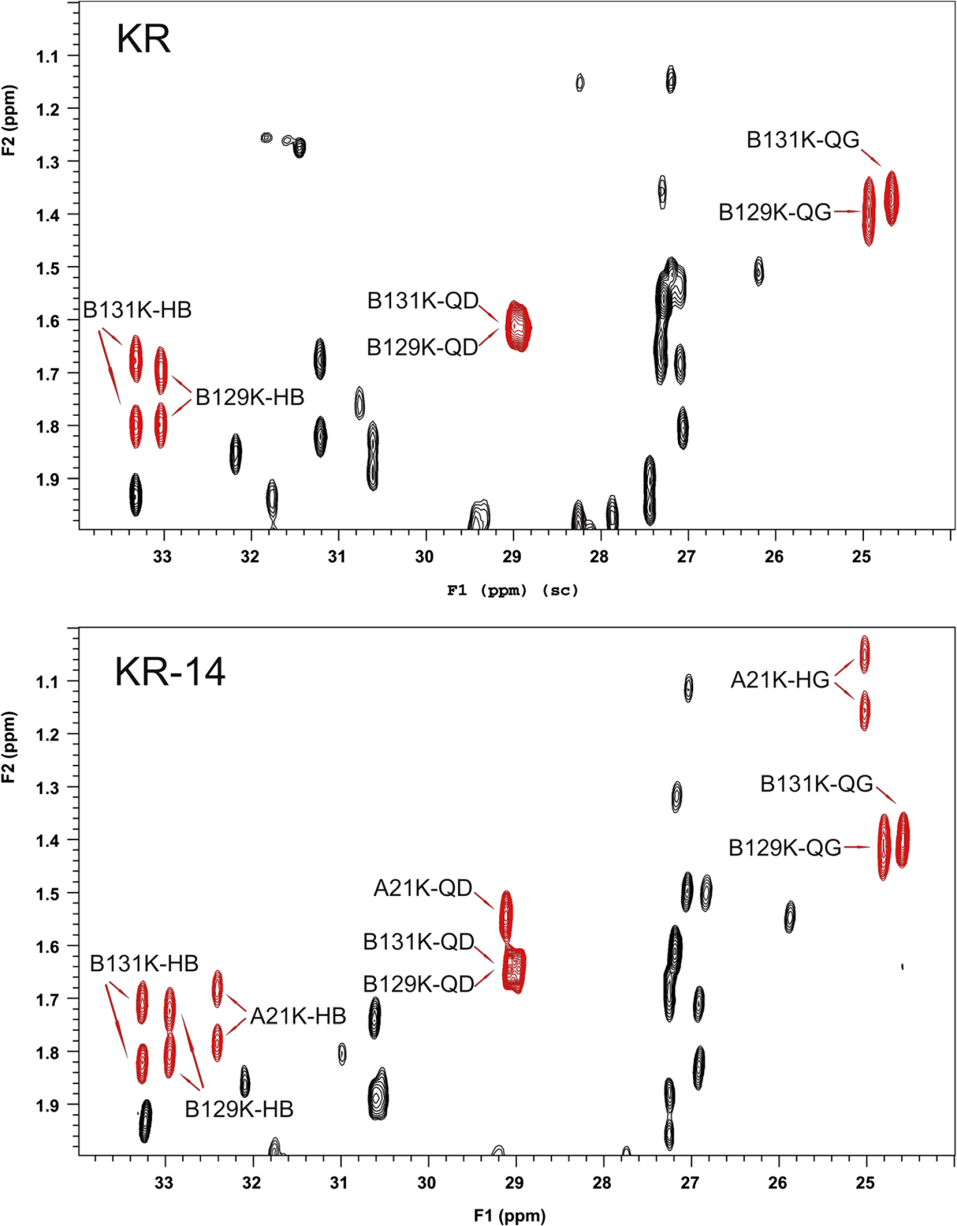
The fragment of ^1^H/^13^C-HSQC spectrum for insulin KR and impurity KR14 contains cross peaks of CH_2_ group from lysine at the β, γ and δ positions (marked in red)

**Fig. 6:**
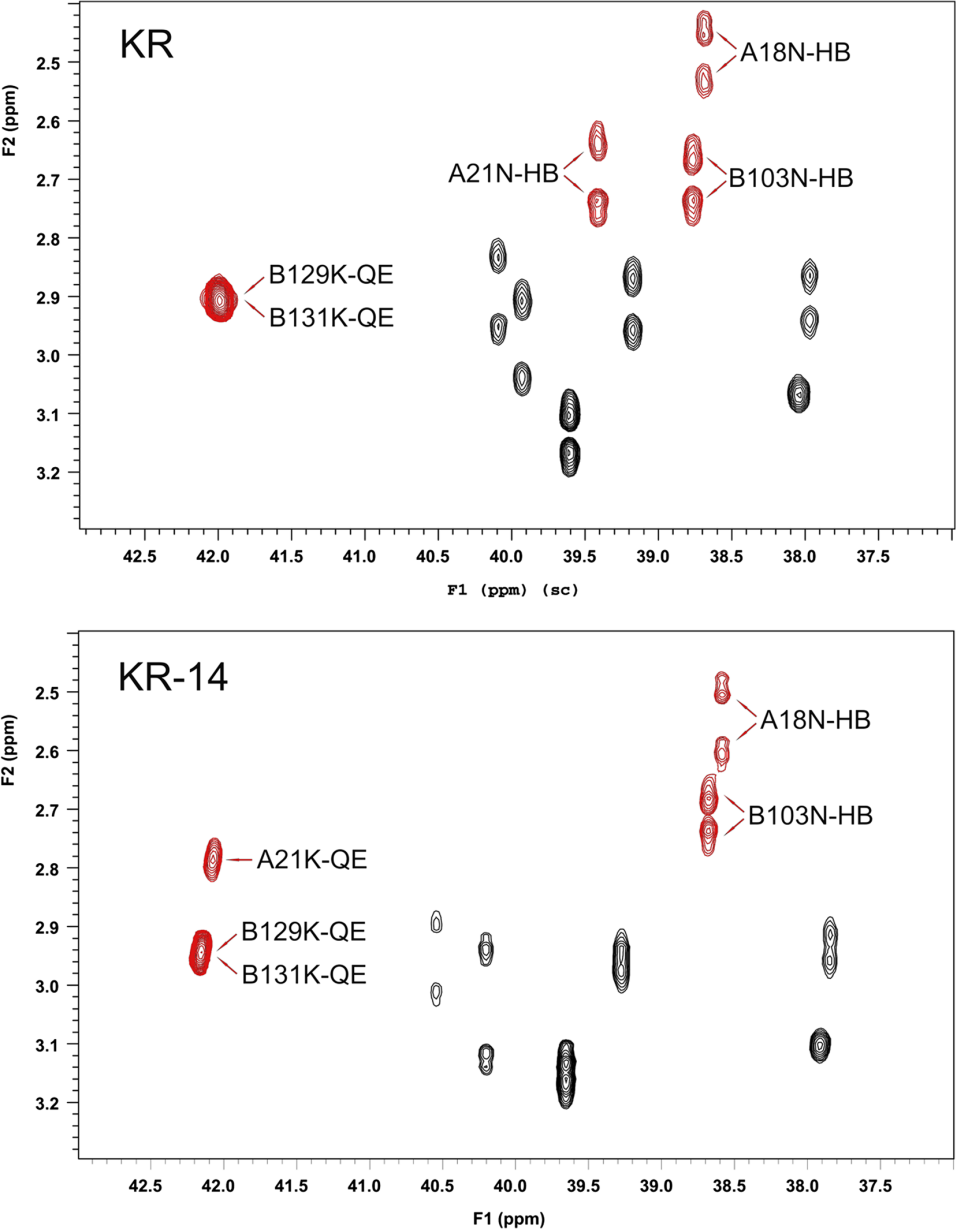
The fragment of ^1^H/^13^C-HSQC spectrum for insulin KR and impurity KR14 contains cross peaks of CH_2_ group from lysine and asparagine at the ε and β positions, respectively (marked in red)

The ^1^H, ^13^C and ^15^N NMR data (chemical shifts, δ, [ppm]) for impurity KR14 are given in Table S1 in Supplementary materials. The TOCSY, ^1^H/^13^C-HSQC and NOESY spectra of the impurity KR14 were compared with the corresponding spectra of insulin KR (32). It was found that the impurity KR14 and insulin KR consist of the same amino acids occurring in the same amount except for differences in the number of lysines (K) and asparagines (N). There are three lysines and two asparagines in the impurity KR14, while in insulin KR – two lysines and three asparagines occur, what can be inferred from ^1^H/^13^C-HSQC spectra presented in Fig. 5 and 6. In the specific ranges for the presence of cross peaks of CH_2_ group from lysine at the β, γ, δ (Fig. 5) and ε positions (Fig. 6), for each of those groups three cross peaks are observed in the spectrum of the impurity KR14 whereas two cross peaks are evident in the spectrum of insulin KR. At the same time in the area of occurrence of cross peaks of CH_2_ group in β position of asparagine (Fig. 6) there are two and three cross peaks, in the spectra of impurity KR14 and insulin KR, respectively.

**Fig. 7:**
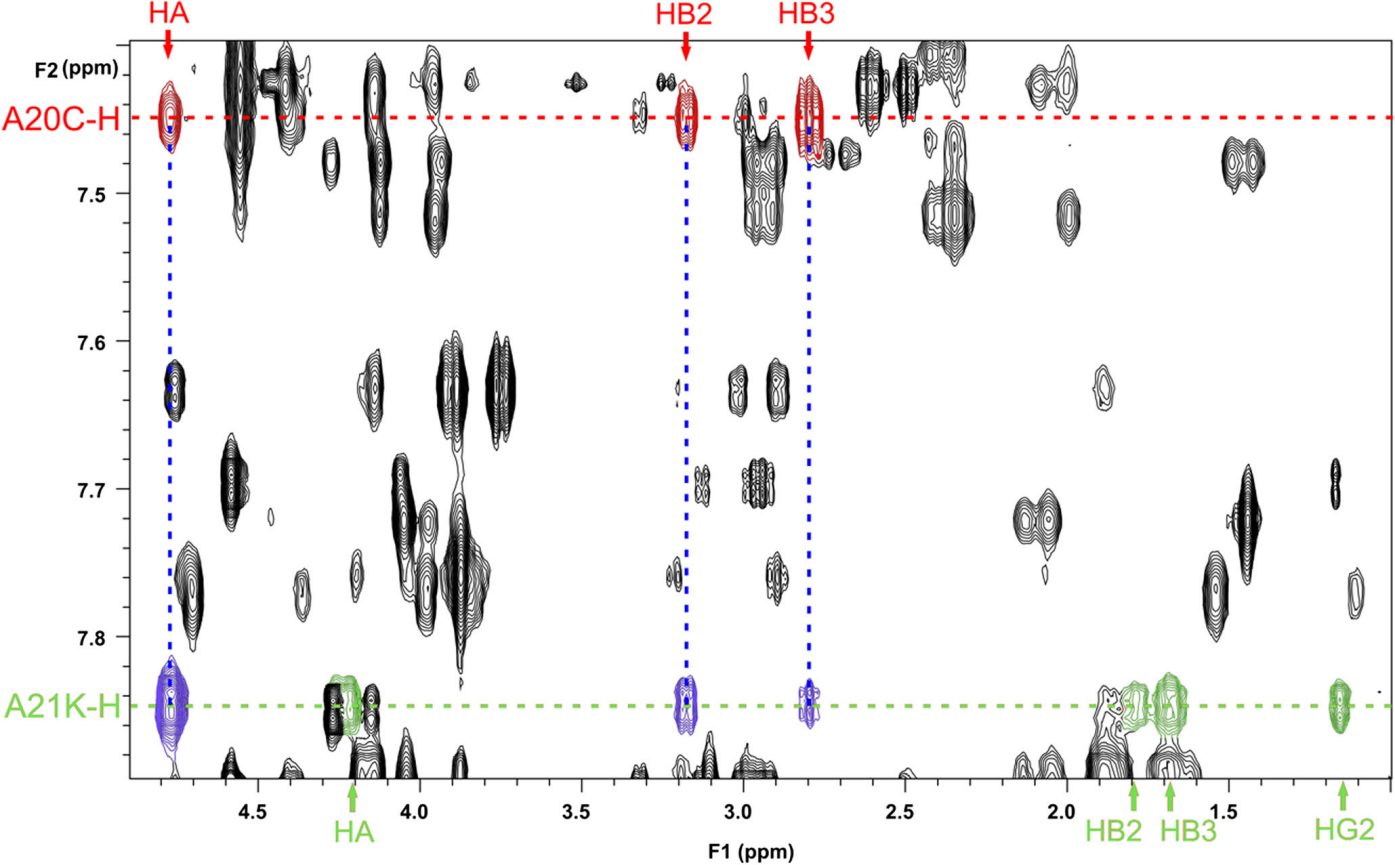
The fragment of NOESY spectrum for impurity KR14

The NOESY spectrum also supports the presence of lysine at A21 in impurity KR14. As it is evident from Fig. 7, there are cross-peaks of Hα and Hβ protons of cysteine at position A20 to NH of the same unit: HαA20-NHA20 (4.78/7.45 ppm), HβA20-NHA20 (3.17/7.45 ppm and 2.79/7.45 ppm) (marked in red) and to NH of the amino acid unit assigned to position A21: HαA20-NHA21 (4.78/7.84 ppm), HβA20-NHA21 (3.17/7.84 ppm and 2.79/7.84 ppm) (marked in blue). The proton signal of the NH group at 7.84 ppm was assigned to one of three lysine residues identified in the structure of the impurity KR14. The cross-peaks of Hα and Hβ protons of lysine A21 to NH of the same unit: HαA21-NHA21 (4.21/7. 84 ppm), HβA21-NHA21 (1.79/7.84 and 1.68/7.84) were marked in green. There are no cross peaks between Hα or Hβ protons of lysine to another NH group, which is an additional proof of lysine A21 at the end of the A chain.

Based on the analysis of the NMR spectra of the impurity KR14, supported by the comparison of the obtained spectra to the spectra of the known structure of insulin KR, it can be confidently stated that the impurity KR14 is Lys^A21^Lys^B31^Arg^B32^ human insulin. Its 3D structure was calculated and compared to insulin KR (32) and human insulin (see Supplementary materials, Fig. S6, Table S2, Fig. S7). The structure of impurity KR14 has all secondary motifs which are present in human insulin (see Fig. S6, Table S2 in Supplementary materials). There are two differences with respect to the tertiary structure of human insulin i.e. there is a deviation in the C-terminus of the A chain, and deviation in the loop region of the B chain of KR14. The higher mobility of the C-terminus of the B chain comparing to the human insulin is also present, but it is analogous to that in insulin KR. The fraying of both mentioned ends is a result of amino acid additions to the B polypeptide chain or modification of the A chain. This may not be critical as the biological activity of a monomer is concerned, however, the oligomerization in water may be affected because structural fluctuations in the interface region can disturb dimerization along the B-chain C-terminus.

### Considerations on Misincorporation

Although the aim of our study was to identify impurity KR14, we were also curious about what would be a possible cause of such misincorporation. It is known that frequency of translation errors increases during overexpression of heterologous proteins in *E.coli* due to stressed conditions, amino acid starvation and translation from nonoptimal codons (20,39,40). Infidelity of the genetic code translation on the molecular level can result from the shift of the mRNA reading frame, tRNA mischarging or codon misreading. The first mentioned mechanism is relatively rare and leads to producing a protein with different characteristics than the desired product. The second and third mechanism might generate a protein derivative that varied with only one amino acid. Mischarging of an incorrect amino acid to the tRNA might be caused by stressed conditions and temporary amino acid starvation (39). Bacteria can incorporate exogenous amino acids when available in preference to synthesizing them from a simple nitrogen and carbon source (41). Precursor-product amino acids of one enzymatic pathway often occupy the contiguous codons. Nevertheless, in an optimized expression system, the misincorporation results mainly from mRNA/tRNA mismatching. This phenomenon is widely investigated and several factors, such as the overall A/U/C/G content, mRNA folding and the polypeptide strand structure might have an impact on the increased probability of the incorrect codon recognition (42,43). However, it is believed that the wobble codon position, that permits non-Watson–Crick base pairing, is responsible for the most cases of amino acid misincorporation (19). The G/U wobble base pair is part of evolutionarily conserved mechanisms that play important, functional role in a wide range of biological processes (44). The guanine-uracil pair is able to form two hydrogen bonds and has the comparable thermodynamic stability to Watson–Crick base pairs.

There are three asparagine residues in human insulin located at B3, A18 and A21 positions. However, only a derivate with misincorporated lysine at A21 was detected. Several insulin analogs with extended A and B chains were investigated to find out why this position is so prone to the misincorporation (Fig. S8 in Supplementary materials). The results showed that neither additional amino acid at A22 nor extended B chain influence this process. In recombinant insulin and its analogs the asparagine residue is coded by two codons: AAC at B3 and A18 and AAU at A21. It clearly indicates that in this case, the misincorporation of lysine at A21 is related to the wobble codon position. Lysine occupies the contiguous codons: AAG and AAA. Therefore it is possible that the lysine misincorporation mechanism is involved with G/U or A/U wobble base pairing.

## Conclusions

Asparagine to lysine misincorporation has been detected and identified in recombinant human insulin and its analogs expressed in *E. coli*. The sequence variants were characterized by peptide mapping, RP-HPLC, MALDI-TOF/TOF mass spectrometry, NMR and Edman sequencing. The misincorporation has been identified at only one out of three Asn sites (A21), exclusively coded by the AAU codon. This indicates a possible correlation between the amino acid substitution and an error-prone codon within the *E. coli* expression system. Furthermore, the lysine misincorporation mechanism may be involved with G/U or A/U wobble base pairing.

### ACKNOWLEDGMENTS AND DISCLOSURES

This study was supported by EU within the European Regional Development Fund (POIG. 01.01.02–00-007/08–04) and by the National Centre for Research and Development within the Applied Research Programme Fund (PBS2/A9/27/2013).

We thank Dr. Paweł Mak from Selvita Ltd., Kraków, Poland for Edman sequencing. The authors declare no competing financial interest.

## Electronic supplementary material

ESM 1 (DOC 1195 kb)

## Abbreviations

CPB: Carboxypeptidase B
IBA: Institute of Biotechnology and Antibiotics, Warsaw, Poland
Insulin KR: Lys^B31^Arg^B32^ human insulin
Insulin KR14: Lys^A21^Lys^B31^Arg^B32^ human insulin
