# Supplementary material for: Identification of Lysine Misincorporation at Asparagine Position in Recombinant Insulin Analogs Produced in E. coli
Source: Pharm Res. 2019 Apr 4;36(6):79. doi: 10.1007/s11095-019-2601-z (PMC6449291; doi:10.1007/s11095-019-2601-z)
Supplement: Supplementary file 1 — (DOC 1195 kb) [file 11095_2019_2601_MOESM1_ESM.doc]

SUPPLEMENTARY MATERIALS TO

**Identification of Lysine Misincorporation at Asparagine Position in Recombinant Insulin Analogs Produced in *E. coli***

Dorota Stadnik*^1^, Anna Bierczyńska-Krzysik^1^, Joanna Zielińska^1^, Jarosław Antosik^1^, Piotr Borowicz^1^, Elżbieta Bednarek^2^, Wojciech Bocian^2^, Jerzy Sitkowski^2^, Lech Kozerski^2^

^1^Institute of Biotechnology and Antibiotics, Starościńska 5, 02-516 Warsaw, Poland

^2^National Medicines Institute, Chełmska 30/34, 00-725 Warsaw, Poland

Correspondence to Dorota Stadnik: stadnikd@iba.waw.pl

**Content of Supplementary Materials**

Descriptions:

- Digestion with CPB.
- Tertiary structure calculation procedure.
- Refinement protocol.

Figures:

- Figure S1. Scheme of manufacturing process of human insulin and its analogues.
- Figure S2. MALDI-TOF/TOF spectrum of Lys^B31^Arg^B32^ human insulin (upper) and the impurity (bottom) cleaved with carboxypeptidase B.
- Figure S3. MALDI-TOF/TOF spectrum of insulin KR (upper) and impurity KR14 (bottom) cleaved with carboxypeptidase B and reduced with DAN matrix.
- Figure S4. MALDI-TOF/TOF spectrum of the fragment II of the impurity KR14 (upper) before, (bottom) after cleavage with carboxypeptidase B.
- Figure S5. HPLC chromatogram of the PTH amino acid residues detected during the 4th cycle of Edman degradation of fragment II of impurity KR14.
- Figure S6. Ribbon drawing of – from left to right – Lys^A21^Lys^B31^Arg^B32^ human insulin, Lys^B31^Arg^B32^ human insulin and human insulin standard.

Figure S7. Average chemical shifts changes for each residue between Lys^A21^Lys^B31^Arg^B32^ human insulin (KR14) and respectively Lys^B31^Arg^B32^ human insulin (KR) – blue and human insulin – red columns.

- Figure S8 MALDI-TOF spectra of enzymatic digests of – from top to bottom – insulin lispro precursor, Ala^A22^Lys^B31^Arg^B32^ human insulin, Gly^A22^Lys^B31^Arg^B32^ human insulin.

Tables:

- Table S1. ^1^H, ^13^C, ^15^N chemical shifts (ppm) of Lys^A21^Lys^B31^Arg^B32^ human insulin (insulin KR14) in H_2_O/ CD_3_CN (73/27 vol.%), 2.9 mM solution, pH 2.3.
- Table S2. Structural statistics of Lys^A21^Lys^B31^Arg^B32^ human insulin (KR14), PDB – 5mwq in comparison to Lys^A21^Lys^B31^Arg^B32^ human insulin (KR), PDB – 2rn5 and human insulin, PDB – 2jv1.

**Digestion with CPB**

Digestion was performed on intact impurity KR14 concurrently with the reference insulin KR. As a result, molecular weight decreased from 6089 to 5804 kDa for insulin KR and from 6103 kDa to 5818 kDa for impurity KR14 (Figure 2S). In both cases, the mass decrease was 285 Da, which corresponds to the loss of one Lys and one Arg residue. For the reference (insulin KR), it can be easily deduced that the (B31) Lys and (B32) Arg were released from the B chain. It is not that obvious for impurity KR14 if the misincorporated Lys is considered. To establish from which chain the hydrolyzed Lys originated, the mass spectra of the impurity KR14 digest were registered with DAN as a MALDI matrix. The same procedure was carried out for the reference sample. DAN is able to activate reduction processes in the laser plume, resulting in disulphide bridge cleavage [1]. Therefore, the signals corresponding to the A and B chains of both analyzed proteins can be distinguished in Figure 3S.

The m/z values for the B chain (3428.5) are the same for both proteins, however, they differ for chain A. The signal registered at m/z 2383.0 for the reference sample is in agreement with theoretical calculations. In the case of impurity KR14, the signal for chain A appears at m/z 2396.9 which is 14 Da more than for insulin KR. This suggests that in both cases Lys and Arg residues were hydrolyzed from the B chain. The A21 residue that was suspected to be misincorporated Lys or Gln was not cleaved off. This indicates the presence of Gln rather than Lys at A21. However, knowing that enzymatic hydrolysis may be affected by structural hindrance [2], [3] digestion with CPB was also carried out on the isolated fragment II. In such a procedure residue A21 from fragment II was easily liberated as can be deducted from the spectra in Figure S4. The difference between the apparent mass of intact and digested fragment II was 128.1 Da which corresponds to the mass of lysine.

**Tertiary structure calculation procedure**

The 1623 NOE cross peaks, 40 coupling constants ^3^*J* (H^α^,NH), 272 ^1^H NMR assigned chemical shifts were used in the automatic NOESY assign procedure, ‘*noeassign’* of CYANA [4]. For an initial step (zero step) of automatic NOESY assignment a structure analogical to X-ray structure of human insulin was used and for further calculation steps. A total of 200 structures of insulin with random geometry were generated by CYANA and refined iteratively using the simulated annealing protocol (10 000 total steps) of the torsion angle dynamics CYANA (version 1.0.6) with optimized chemical shift list tolerance of 0.022/0.022 ppm. This gave 715 upper bound restraints and 498 angle restraints. The 100 structures with lowest CYANA target function values were used for further MD refinement in AMBER 15 [5]. In AMBER calculations additionally to the 715 distance restraints and selected 498 torsions *ϕ* and *ψ,* 9 disulfides, 131 chirality and 51 trans- *ω* restraints were used. The Generalized Born (GB) solvent model [6], [7] approach was used in the refinement protocol using molecular dynamics simulated annealing.

***Refinement protocol***

The 500 steps energy minimization with GB model was first performed on the CYANA structures, followed by two cycles of 15 ps simulated annealing with GB were performed by *pmemd* (from AMBER): 0-1000 steps heating the system from 10 to 1100 K, 1001-3000 steps leaving at 1100K, 3000-15000 steps cooling to 0 K, 0-3000 steps tight coupling for heating and equilibration (TAUTP = 0.2), 3001-11000 steps of slow cooling (TAUTP = 4.0-2.0), 11000-13000 steps of faster cooling (TAUTP = 1.0) and 13000-15000 steps of fast cooling, like a minimization (TAUTP = 0.5-0.05). In steps 0-3000 the restraints were slowly increased from 10% to 100% of its final values. A cutoff value was set to 15 Å. For further analysis from 100 refined structures, 50 structures were selected with the lowest violation energy and, next from these, final 20 structures with the lowest total AMBER energy were chosen as structure representatives. FigureS-3 shows an ensemble of 20 calculated structures of lowest energy of impurity KR14 in comparison to insulin KR and human insulin [8].


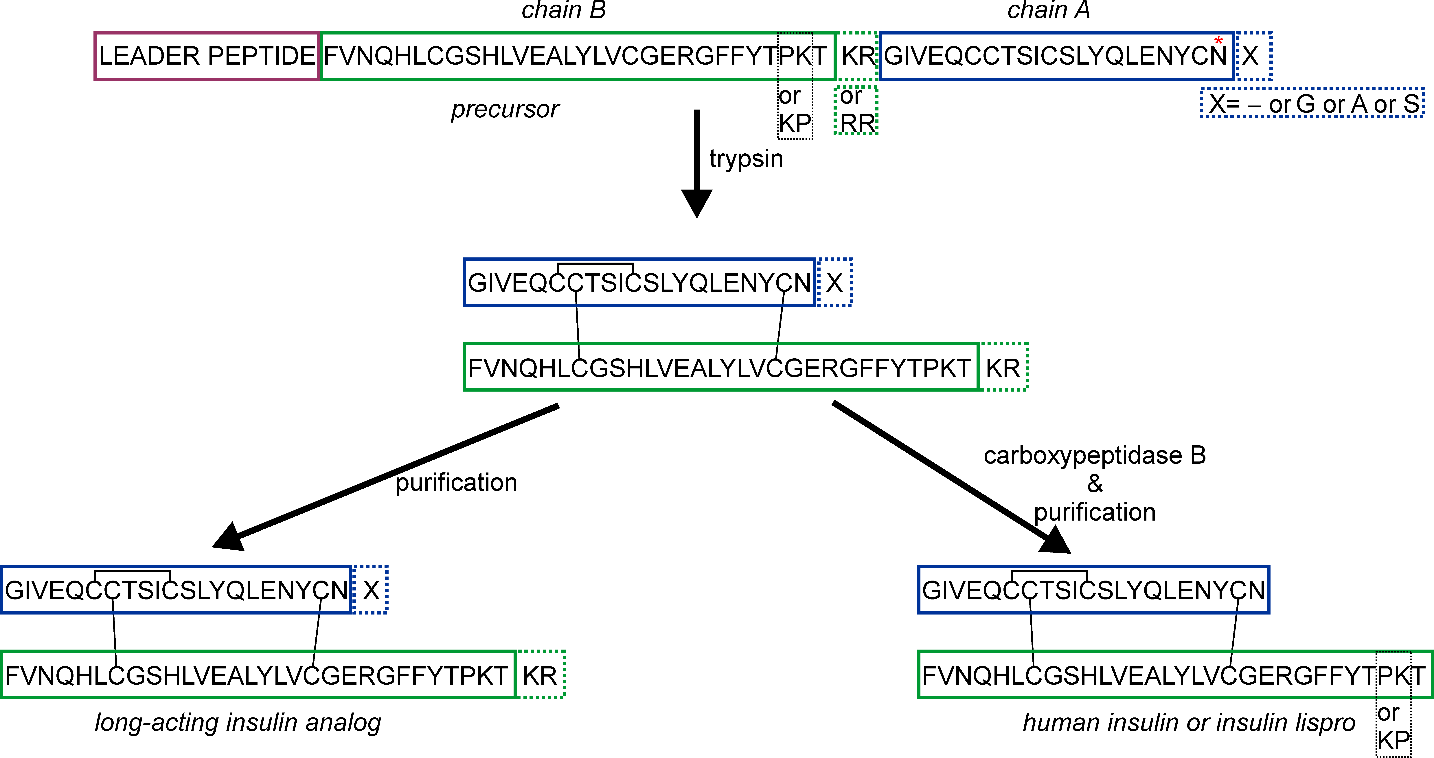


Figure S1. Scheme of manufacturing process of human insulin and its analogs; the red dot indicates the residue prone to Asn→Lys misincorporation.


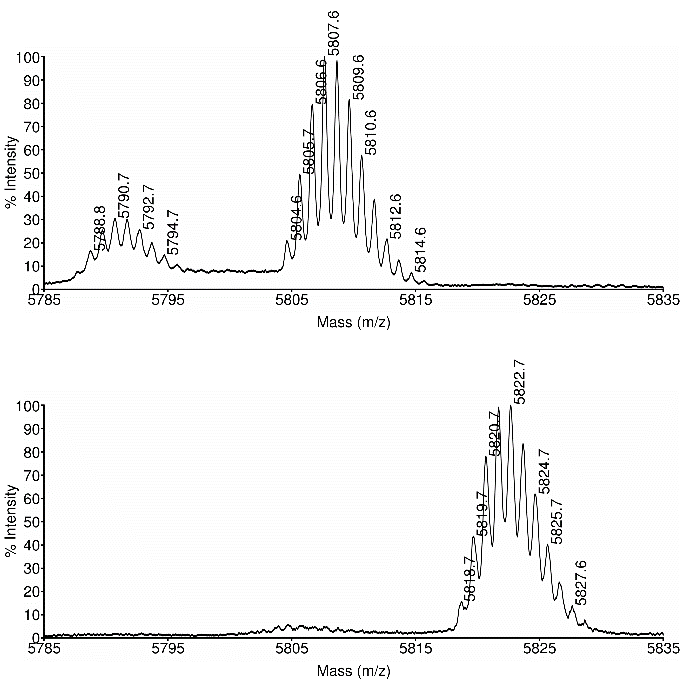


Figure S2. MALDI-TOF/TOF spectrum of Lys^B31^Arg^B32^ human insulin (upper) and the impurity (bottom) cleaved with carboxypeptidase B.


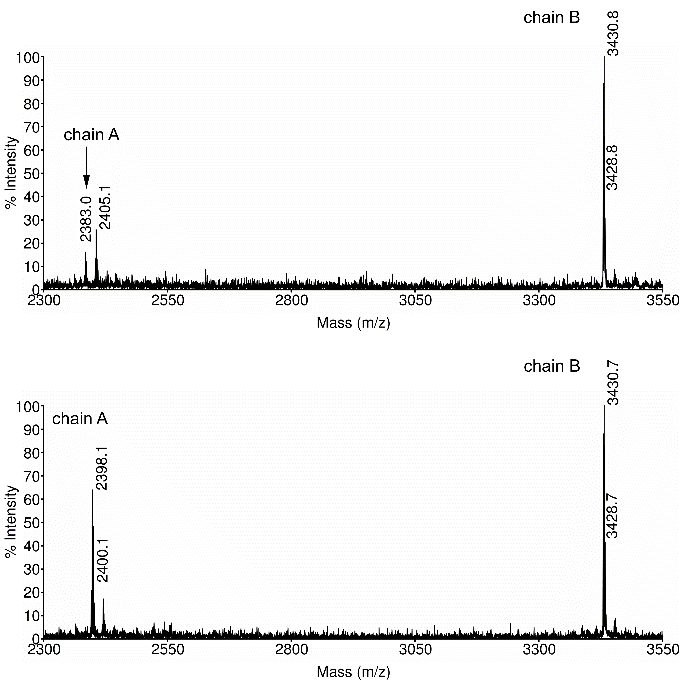


Figure S3. MALDI-TOF/TOF spectrum of insulin KR (upper) and impurity KR14 (bottom) cleaved with carboxypeptidase B and reduced with DAN matrix.


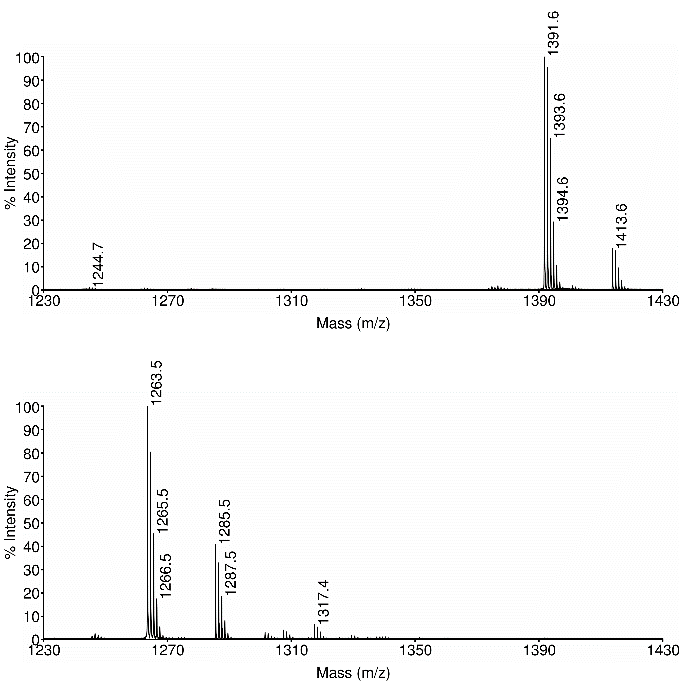


Figure S4. MALDI-TOF/TOF spectrum of the fragment II of the impurity KR14 (upper) before, (bottom) after cleavage with carboxypeptidase B.


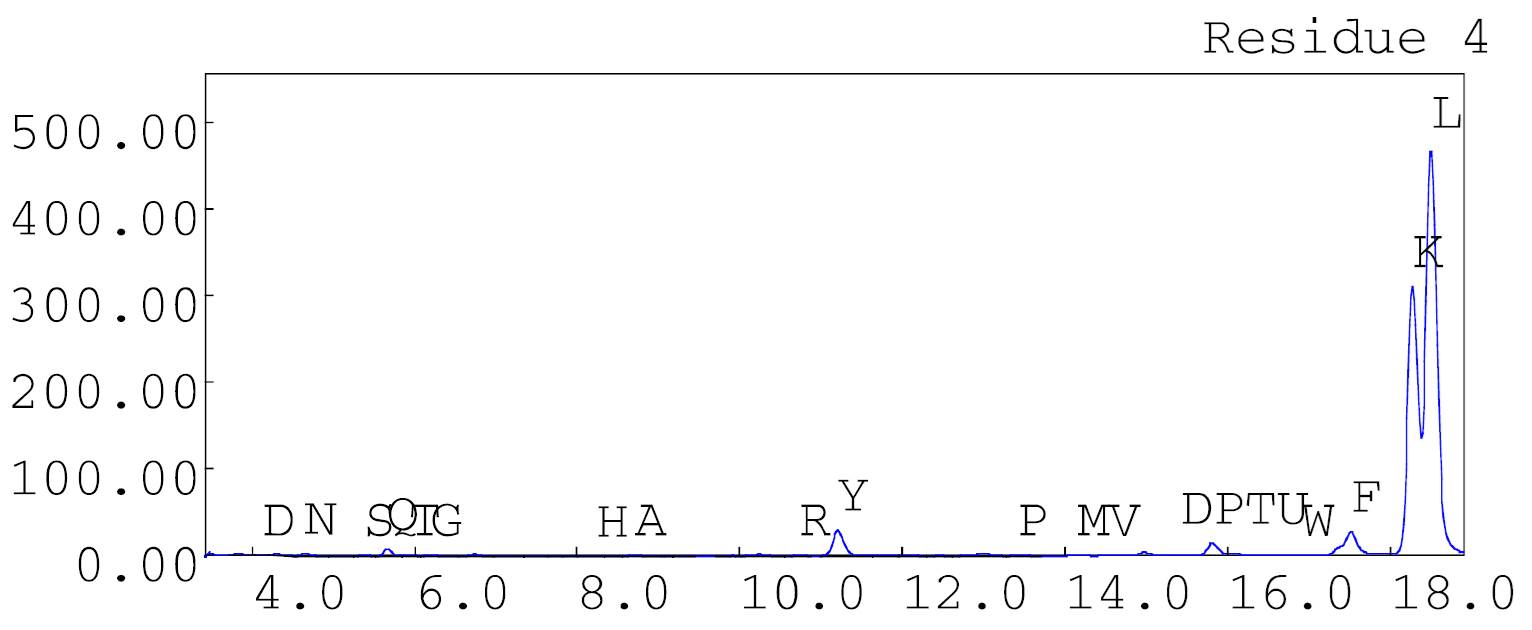


Figure S5. HPLC chromatogram of the PTH amino acid residues detected during the 4th cycle of Edman degradation of fragment II of impurity KR14.


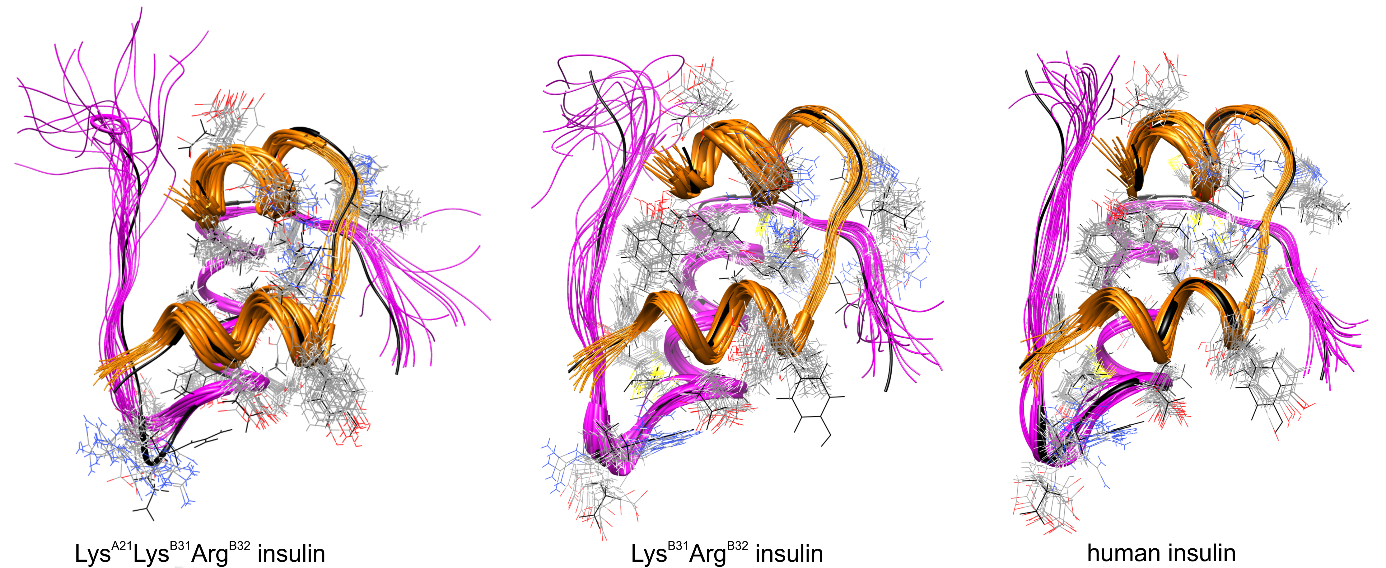


Figure S6. Ribbon drawing of – from left to right – Lys^A21^Lys^B31^Arg^B32^ human insulin, Lys^B31^Arg^B32^ human insulin and human insulin standard; chain A in orange, chain B in magenta; black string represent the X-ray structure of human insulin.

**
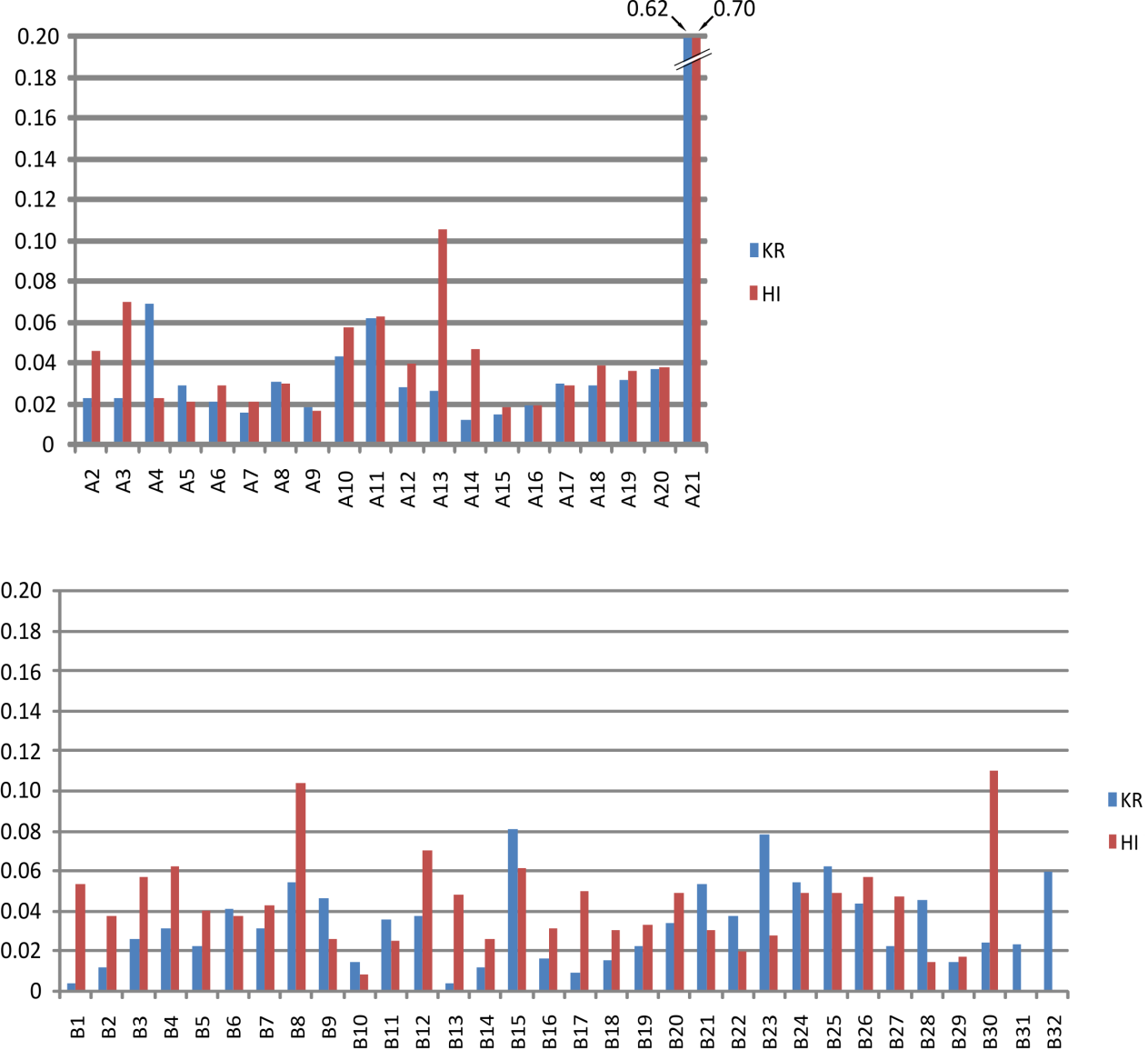
**

Figure S7. Average chemical shifts changes for each residue between Lys^A21^Lys^B31^Arg^B32^ human insulin (KR14) and respectively Lys^B31^Arg^B32^ human insulin (KR) – blue and human insulin (HI) – red columns.

Figure S7 shows average over each residue chemical shifts changes between impurity KR14 and insulin KR or human insulin. The biggest change is observed for residue A20 what is obvious due to substitution of A21. The rest of rather moderate changes of chemical shifts in the A chain and partly in the B chain may possibly by associated with changes in the dynamics of this regions of amino acid chains. The quite big chemical shifts changes in B15 and B23-B24 regions can be, however, associated with steric interaction of amended A21 amino acid.


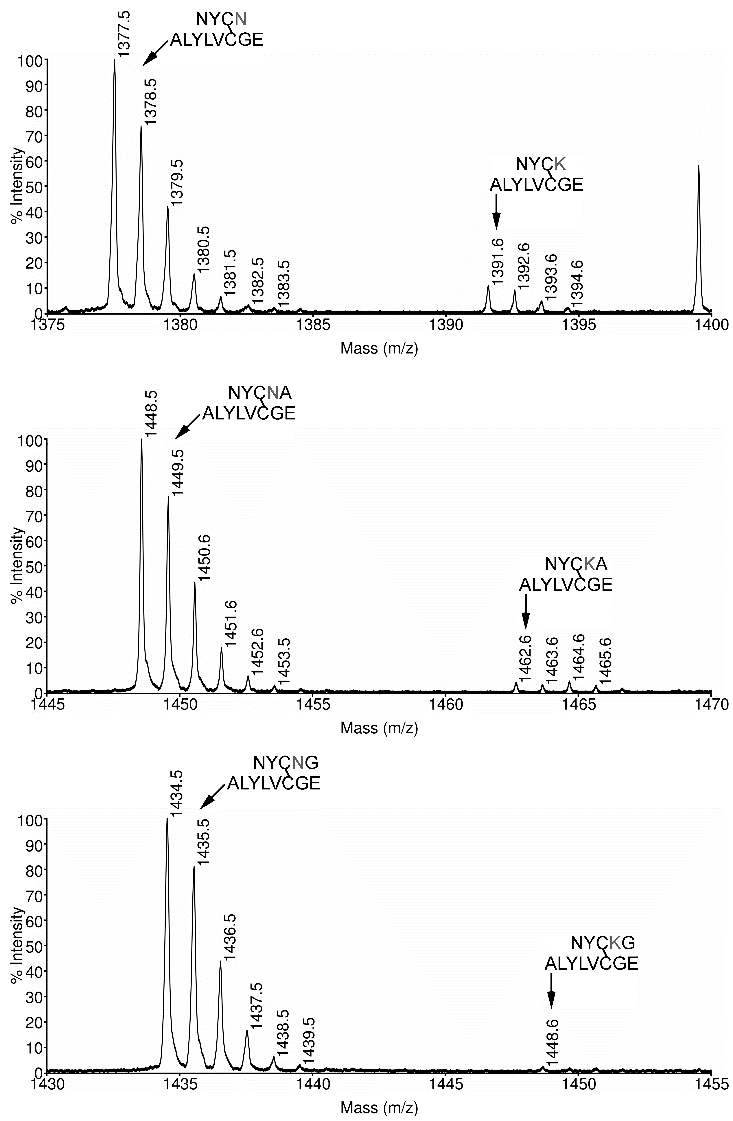


Figure S8. MALDI-TOF spectra of enzymatic digests of – from top to bottom – insulin lispro precursor, Ala^A22^Lys^B31^Arg^B32^ human insulin, Gly^A22^Lys^B31^Arg^B32^ human insulin.

Table S1. ^1^H, ^13^C, ^15^N chemical shifts (ppm) of Lys^A21^Lys^B31^Arg^B32^ human insulin (insulin KR14) in H_2_O/ CD_3_CN (73/27 vol.%), 2.9 mM solution, pH 2.3.

| Res |  | NH | | **HA** | **CA** | HB | | HB2 | **HB3** | CB | HG | | HG2 | **HG3** | | **CG** | **Other** |
| --- | --- | --- | --- | --- | --- | --- | --- | --- | --- | --- | --- | --- | --- | --- | --- | --- | --- |
|  |  | H | **N** |  |  |  |  |  |  |  |  |  |  |  |  |  |  |
| A1 G | H | **--** | **--** | **3.94** | **43.46** | **--** | | | | **--** | **--** | | | | | **--** | **--** |
| A2 I | CH(CH_3_)-CH_2_-CH_3_ | **8.414** | **121.23** | **3.938** | **62.8** | **1.332** | **--** | | | **38.41** | **HG11 1.189 HG12 0.960**  **QG2 0.758** | | | | | **CG1 28.04**  **CG2 16.91** | **QD1 0.640; CD1 14.00** |
| A3 V | CH(CH_3_)_2_ | **8.007** | **123.19** | **3.682** | **65.10** | **1.990** | **--** | | | **31.76** | **QG1 0.928**  **QG2 0.874** | | | | | **CG1 22.12**  **CG2 21.18** | **--** |
| A4 E | CH_2_CH_2_COOH | **8.110** | **121.87** | **4.230** | **n.o.** | **--** | **QB 2.050** | | | **28.11** | **--** | **QG 2.468** | | | | **33.08** | **--** |
| A5 Q | CH_2_CH_2_CONH_2_ | **8.280** | **n.o.** | **4.01** | **58.83** | **--** | **2.079** | | **2.008** | **29.25** | **---** | **2.430** | | | **2.350** | **33.97** | **HE21 7.471; HE22 6.786** |
| A6 C | CH_2_SH | **8.183** | **111.28** | **4.840** | **54.68** | **--** | **3.278** | | **2.840** | **n.o.** | **--** | | | | | **--** | **--** |
| A7 C | CH_2_SH | **8.247** | **118.44** | **4.776** | **57.22*** | **--** | **3.700** | | **3.270** | **39.22** | **--** | | | | | **--** | **--** |
| A8 T | CH(OH)-CH_3_ | **8.098** | **112.91** | **4.023** | **64.56** | **4.35** | **--** | | | **68.66** | **QG2 1.208** | | | | | **CG2 22.27** | **--** |
| A9 S | CH2OH | **7.420** | **116.83*** | **4.699** | **n.o.** | **--** | **3.976** | | **3.830** | **64.13** | **--** | | | | | **--** | **--** |
| A10 I | CH(CH_3_)-CH_2_-CH_3_ | **7.770** | **117.60** | **4.361** | **60.06** | **1.55** | **--** | | | **39.30** | **HG11 1.120; HG12 0.490**  **QG2 0.640** | | | | | **CG1 27.03**  **CG2 17.45** | **QD1 0.540; CD1 12.70** |
| A11 C | CH_2_SH | **9.479** | **n.o.** | **4.980** | **53.03** | **--** | **3.237** | | **3.108** | **n.o.** | **--** | | | | | **--** | **--** |
| A12 S | CH_2_OH | **8.586** | **116.19** | **4.557** | **n.o.** | **--** | **4.271** | | **3.95** | **65.83** | **--** | | | | | **--** | **--** |
| A13 L | CH_2_-CH(CH_3_)_2_ | **8.638** | **121.83** | **3.924** | **58.43** | **--** | **1.49** | | **1.43** | **41.38** | **1.497** | **--** | | | | **27.05** | **QD1 0.858; QD2 0.782**  **CD1 24.16; CD2 24.58** |
| A14 Y | CH_2_-Ph-OH | **7.477** | **115.68** | **4.121** | **60.34** | **--** | **2.96** | | **2.92** | **37.84** | **--** | | | | | **--** | **QD 7.056; QE 6.817** |
| A15 Q | CH_2_-CH_2_-CONH_2_ | **7.514** | **117.60** | **3.959** | **58.64** | **--** | **2.342** | | **1.996** | **29.18** | **--** | **2.413** | | | **2.342** | **35.13** | **HE21 7.405; HE22 6.873** |
| A16 L | CH_2_-CH(CH_3_)_2_ | **8.004** | **119.84** | **4.134** | **58.29** | **--** | **1.891** | | **1.558** | **42.08** | **1.712** | **--** | | | **--** | **26.92** | **QD1 0.795; QD2 0.752**  **CD1 25.69; CD2 24.92** |
| A17 E | CH_2_-CH_2_-COOH | **8.057** | **114.41** | **4.149** | **57.87** | **--** | **2.086** | | **2.010** | **27.74** | **---** | **2.553** | | | **2.328** | **33.52** | **--** |
| A18 N | CH_2_-CONH_2_ | **7.426** | **116.83*** | **4.418** | **n.o.** | **--** | **2.610** | | **2.490** | **38.58** | **--** | | | | | **--** | **HD21 7.151; HD22 6.412** |
| A19 Y | CH_2_-Ph-OH | **7.909** | **116.57** | **4.39** | **59.52** | **--** | **3.321** | | **2.970** | **38.26** | **--** | | | | | **--** | **QD 7.313; QE 6.728** |
| A20 C | CH_2_-SH | **7.45** | **115.81** | **4.78** | **53.76** | **--** | **3.170** | | **2.792** | **36.33** |  | | | | |  | **--** |
| **A21 K** | CH_2_-CH_2-_CH_2_-CH_2_-N^+^H_3_ | **7.844** | **120.97** | **4.214** | **55.79** | **--** | **1.788** | | **1.678** | **32.41** | **--** | **1.160** | | | **1.048** | **25.02** | **QD 1.548; CD 29.12**  **QE 2.78; CE 42.07**  **NH_3_  7.380** |

| **Res** |  | NH | | **H**A | **CA** | **HB** | **HB2** | **HB3** | **CB** | **HG** | **HG2** | HG3 | **CG** | Other |
| --- | --- | --- | --- | --- | --- | --- | --- | --- | --- | --- | --- | --- | --- | --- |
|  |  | H | **N** |  |  |  |  |  |  |  |  |  |  |  |
| B101 F | CH_2_-Ph | **--** | **--** | **4.260** | **57.07** | **--** | **QB 3.15** | | **39.65** | **--** | | | **--** | **QD 7.24; QE 7.36;** |
| B102 V | CH(CH_3_)_2_ | **8.092** | **123.24** | **4.080** | **61.860** | **1.932** | **--** | | **33.23** | **QG1 0.860**  **QG2 0.850** | | | **20.45**  **21.00** | **--** |
| B103 N | CH_2_-CONH_2_ | **8.290** | **122.86** | **4.630** | **n.o.** | **--** | **2.740** | **2.672** | **38.68** | **--** | | | **--** | **HD21 7.471; HD22 6.786** |
| B104 Q | CH_2_-CH_2_-CONH_2_ | **8.226** | **119.54** | **4.387** | **55.22** | **--** | **2.080** | **1.800** | **30.98** | **----** | **2.230** | **2.160** | **33.63** | **HE21 7.349; HE22 6.651** |
| B105 H | CH_2_C_3_H_3_N_2_ | **8.432** | **118.98** | **4.412** | **n.o.** | **--** | **3.524** | **3.224** | **28.45** | **--** | | | **--** | **HD2 7.341; HE1 8.502** |
| B106 L | CH_2_-CH(CH_3_)_2_ | **8.838** | **125.89** | **4.444** | **n.o.** | **--** | **1.689** | **0.910** | **44.71** | **1.548** | **--** | **--** | **25.87** | **QD1 0.840; QD2 0.713**  **CD1 25.96; CD2 23.72** |
| B107 C | CH_2_SH | **8.196** | **118.58** | **4.900** | **54.15** | **--** | **3.190** | **2.942** | **36.63*** | **--** | | | **--** | **--** |
| B108 G | H | **8.955** | **111.67** | **HA2 3.964**  **HA3 3.772** | **46.75** | **--** | | | **--** | **--** | | | **--** | **--** |
| B109 S | CH_2_OH | **8.962** | **121.99** | **4.075** | **61.10** | **3.846** | **--** | **--** | **62.55** | **--** | | | **--** | **--** |
| B110 H | CH_2_C_3_H_3_N_2_ | **7.956** | **118.88** | **4.461** | **n.o.** | **--** | **3.517** | **3.238** | **28.02** | **--** | | | **--** | **HD2 7.423; HE1 8.640** |
| B111L | CH_2_-CH(CH_3_)_2_ | **7.048** | **121.34** | **3.972** | **57.54** | **--** | **1.851** | **1.210** | **40.29** | **1.320** | **--** | **--** | **27.17** | **QD1 0.776; QD2 0.710**  **CD1 25.33; CD2 22.55** |
| B112V | CH(CH_3_)_2_ | **7.228** | **117.44** | **3.325** | **66.74** | **2.034** | **--** | | **31.66** | **QG1 0.945**  **QG2 0.918** | | | **CG1 22.31**  **CG2 21.37** | **--** |
| B113E | CH_2_-CH_2_-COOH | **7.920** | **119.76** | **4.051** | **58.78** | **--** | **2.130** | **2.060** | **27.91** | **QB 2.510** | | | **32.69** | **--** |
| B114A | CH_3_ | **7.721** | **120.67** | **4.051** | **55.35** | **QB 1.440** | | | **18.98** | **--** | | | **--** | **--** |
| B115L | CH_2_-CH(CH_3_)_2_ | **8.050** | **117.46** | **3.890** | **57.72** | **--** | **1.441** | **1.020** | **41.31** | **1.498** | **--** | **--** | **26.83** | **QD1 0.668; QD2 0.490**  **CD1 24.13; CD2 25.53** |
| B116Y | CH_2_-Ph-OH | **8.153** | **119.43** | **4.200** | **61.45** | **QB 3.104** | | | **37.91** | **--** | | | **--** | **QD 7.100; QE 6.734** |
| B117L | CH_2_-CH(CH_3_)_2_ | **7.92** | **116.88** | **4.04** | **57.70** | **--** | **1.892** | **1.621** | **42.27** | **1.832** | **--** | **--** | **26.92** | **QD1 0.928; QD2 0.899**  **CD1 24.99; CD2 23.48** |
| B118V | CH(CH_3_)_2_ | **8.438** | **116.50** | **3.80** | **65.39** | **2.080** | **--** | | **32.37** | **QG1 0.999**  **QG2 0.860** | | | **CG1 22.50**  **CG2 21.55** | **--** |
| B119C | CH_2_SH | **8.592** | **115.63** | **4.730** | **54.44** | **--** | **3.218** | **2.898** | **36.63*** | **--** | | | **--** | **--** |
| B120G | H | **7.757** | **108.48** | **3.872** | **46.37** | **--** | | | **--** | **--** | | | **--** | **--** |
| B121E | CH_2_-CH_2_-COOH | **8.24** | **120.55** | **4.18** | **56.81** | **--** | **2.141** | **2.051** | **28.14** | **QB 2.480** | | | **32.91** | **--** |
| B122R | CH_2-_CH_2_-CH_2_-NH-C^+^(NH_2_)_2_ | **7.892** | **118.82** | **4.136** | **57.19** | **QB 1.883** | | | **30.53** | **QG 1.680** | | | **27.25** | **QD 3.192; CD 43.61**  **HE 7.118** |
| B123G | H | **7.632** | **104.83** | **HA2 3.900**  **HA3 3.746** | **45.07** | **--** | | | **--** | **--** | | | **--** | **--** |
| B124F | CH_2_-Ph | **7.632** | **116.54** | **4.754** | **57.22*** | **--** | **3.016** | **2.892** | **40.54** | **--** | | | **--** | **QD 6.961; QE 7.101** |
| B125F | CH_2_-Ph | **8.174** | **119.00** | **4.586** | **n.o.** | **--** | **3.108** | **2.942** | **40.20** | **--** | | | **--** | **QD 7.201; QE 7.309** |
| B126Y | CH_2_-Ph-OH | **7.906** | **117.94** | **4.589** | **n.o.** | **QB 2.964** | | | **39.29** | **--** | | | **--** | **QD 7.057; QE 6.753** |
| B127T | CH(OH)-CH_3_ | **7.696** | **117.43** | **4.571** | **n.o.** | **4.058** | **--** | | **70.02** | **QG2 1.168** | | | **21.30** | **--** |
| B128P | C_3_H_6_ | **--** | **--** | **4.276** | **63.0** | **--** | **2.174** | **1.866** | **32.10** | **--** | **1.958** | **1.882** | **27.25** | **QD 3.62; CD 50.83** |
| B129K | CH_2_-CH_2-_CH_2_-CH_2_-N^+^H_3_ | **8.167** | **120.60** | **4.261** | **56.44** | **--** | **1.800** | **1.720** | **32.95** | **QG 1.418** | | | **24.80** | **QD 1.648; CD 28.97**  **QE 2.95; CE 42.15;**  **NH_3_ 7.439** |
| B130T | CH(OH)-CH_3_ | **7.848** | **114.17** | **4.273** | **61.660** | **4.150** | **--** | | **69.91** | **QG2 1.154** | | | **21.59** | **--** |
| B131K | CH_2_-CH_2-_CH_2_-CH_2_-N^+^H_3_ | **8.092** | **122.67** | **4.321** | **56.10** | **--** | **1.821** | **1.710** | **33.27** | **QG 1.403** | | | **24.59** | **QD 1.648; CD 29.07**  **QE 2.951; CE 42.15;**  **NH_3_  7.440** |
| B132R | CH_2-_CH_2_-CH_2_-NH-C^+^(NH_2_)_2_ | **8.213** | **122.83** | **4.330** | **55.23** | **--** | **1.892** | **1.738** | **30.61** | **QG 1.610** | | | **27.19** | **QD 3.160; CD 43.34**  **HE 7.142** |

n.o. - not observed

Table S2. Structural statistics of Lys^A21^Lys^B31^Arg^B32^ human insulin (KR14), PDB – 5mwq in comparison to Lys^B31^Arg^B32^ human insulin (KR), PDB – 2rn5 and human insulin, PDB – 2jv1

|  | AMBER_GBa  Lys^A21^Lys^B31^Arg^B32^ human insulin (KR14) | AMBER_GBb  LysB^31^Arg^B32^ human insulin (KR) | AMBER_GBc human insulin |
| --- | --- | --- | --- |
| **Experimental NOE’s**d | 1623 | 1451 | 1251 |
| **Experimental restraints**e | | | |
| Total inter- proton | 715 | 815 | 680 |
| Intra-residue | 219 | 195 | 177 |
| Sequential | 223 | 289 | 251 |
| Medium range | 156 | 188 | 142 |
| Long - range ( /j-i/ > 5) | 117 | 143 | 110 |
| Disulfide restraints | 9 | 9 | 9 |
| Chirality restraints | 131 | 53 | 51 |
| Trans - (ω ) restraints | 51 | 52 | 50 |
| Torsion restraints (ψ and ϕ ) | 498 | 255 | 72 (φ) |
| **Number of distance restraint violations in calculated structures per model**f | | | |
| Total | 28.0 (0.039± 0.014) | 18.3 (0.053± 0.021) | 34.4 (0.057±0.028) |
| Intra -residue | 2.56 (0.049±0.029) | 3.8 (0.070±0.032) | 4.7 (0.069±0.037) |
| Sequential | 7.10 (0.040±0.014) | 5.3 (0.046±0.019) | 19.2 (0.057±0.029) |
| Medium range | 9.03 (0.033±0.007) | 5.2 (0.050±0.012) | 7.2 (0.047±0.011) |
| Long range | 9.30 (0.040±0.010) | 4.1 (0.049±0.012) | 3.2 (0.061±0.025) |
| **RMSD from mean structure**g | | | |
| All atoms in ensemble | 2.60 (1.18) | 2.50 (1.50) | 1.828 (1.265) |
| Backbone heavy atoms | 1.62 (0.47) | 1.47 (0.73) | 0.991 (0.482) |
| Long range restraints |  |  | 0.8883 |
| **RMSD fromX-Ray structure**h | | | |
| All atoms in ensemble | (2.01) | (2.34) | 2.526 (1.814) |
| Backbone heavy atoms | (0.92) | (0.96) | 1.313 (0.703) |
| Long range restraints |  |  | 1.3718 |
| **Ramachandran statistics ( % residues included in )** | | | |
| Most favored regions | 87.4 | 89.8 | 92.4 |
| Additionally allowed regions | 10.4 | 8.2 | 6.9 |
| Generously allowed regions | 0.7 | 1.2 | 0.2 |
| Disallowed regions | 1.4 | 0.8 | 0.5 |

1. Insulin KR14 in solvent, structure deposited in PDB – 5mwq.
2. Insulin KR in solvent, structure deposited in PDB – 2rn5.
3. Human insulin in solvent, structure deposited in PDB – 2jv1, BRMB accession No. 15464.
4. The numbers of considered NOE cross peaks. Only unambiguous cross peaks were used in input for human insulin. The number of automatically assigned cross peaks for KR14 is 1405.
5. Transposed by CYANA to distance restraints.
6. The 715 constraints in insulin KR14 and 815 constraints in insulin KR (both in 100 models), and 680 constraints considered in human insulin (in 200 models). Values in parentheses show average violation, Å.
7. 20, 50 and 50 low energy structures are compared for insulin KR14, insulin KR, and human insulin, respectively. Values in parentheses refer to statistic with excluded ultimate units in both chains, i.e.; residues 2-20 and 3-29 in chain A and B, respectively for human insulin and KR, but 3-20 and 5-20 residues in insulin KR14.
8. Human insulin, X-ray structure deposited in PDB – 1mso.

**References:**

1. Molin L, Seraglia R, Dani FR, Moneti G, Traldi P. The double nature of 1,5-diaminonaphthalene as matrix-assisted laser desorption/ionization matrix: some experimental evidence of the protonation and reduction mechanisms. Rapid Commun Mass Spectrom. 2011;25(20):3091–6.

2. Zappacosta F, Pessi A, Bianchi E, Venturini S, Sollazzo M, Tramontano A, et al. Probing the tertiary structure of proteins by limited proteolysis and mass spectrometry: The case of minibody. Protein Sci. 2008;5(5):802–13.

3. Kemmler W, Peterson JD, Steiner DF. Studies on the conversion of proinsulin to insulin. I. Conversion in vitro with trypsin and carboxypeptidase B. J Biol Chem. 1971;246(22):6786–91.

4. Güntert P, Mumenthaler C, Wüthrich K. Torsion angle dynamics for NMR structure calculation with the new program DYANA. J Mol Biol. 1997;

5. Case D., Berryman JT, Betz RM, Cerutti DS, Cheatham III TE, Darden TA, et al. 2015, AMBER 2015, University of California, San Francisco.

6. Onufriev A, Bashford D, Case DA. Modification of the Generalized Born Model Suitable for Macromolecules. J Phys Chem B. 2000;104(15):3712–20.

7. Xia B, Tsui V, Case DA, Dyson HJ, Wright PE. Comparison of protein solution structures refined by molecular dynamics simulation in vacuum, with a generalized Born model, and with explicit water. J Biomol NMR. 2002; 22(4), 317–331.

8. Bocian W, Borowicz P, Mikołajczyk J, Sitkowski J, Tarnowska A, Bednarek E, et al. NMR structure of biosynthetic engineered human insulin monomer B31 ^Lys^ -B32 ^Arg^ in water/acetonitrile solution. Comparison with the solution structure of native human insulin monomer. Biopolymers. 2008;89(10):820–30.
